# Face Presentations

**Published:** 1890-04

**Authors:** J. J. Green

**Affiliations:** Pittsburgh, Pa.


### FACE PRESENTATIONS.*

BY J. J. GREEN, M. D., PITTSBURG, PA.

We understand by a face presentation that the chin is extended, that the occiput is reflected against the neck, and the face, with the frontal portion of the skull, occupies, or has a great tendency to occupy, the pelvic entrance. This condition does not occur frequently. Statistics show that among French obstetricians, about one in 250 presents a face. Dr. Churchill, some years ago, found that in British practice, face presentations occurred once in 292 cases.

According to German statistics, it occurs once in 130 cases. If it could be proven that the difference in number really occurs, we might conclude that some difference in the anatomical formation of the pelvis exists, or that the fœtal head among Germans is somewhat less; or may more frequently possess a long occiput.

Face presentations seem to solicit only a cursory mention by many of our writers in obstetrics. We regard the subject of much more importance than seems to be attached to it by many

* Read at Pittsburgh Obstetrical Society, Nov. 7th, 1889

writers. When we review the causes leading to face presentations, and difference of opinion with reference to the management of same, it will be noticed that some good authorities recommend one method of procedure, while equally as good advocate manipulation almost directly opposite. One directs version, another non-interference. Doctrines of this character tend largely to confuse the general practitioners—the men who really occupy the front rank among obstetricians, for it is they who wait on the legitimate mothers of families.

Edward L. Partridge asserts that there is no great disparity in the views of obstetricians upon the cause of face presentations. This may be true in a measure; yet it is equally true that scarcely any one writer coincides in a majority of particulars with any one else.

There seems little dispute about the classes or varieties of face presentations. Anterior, transverse, and the posterior varieties, we think, may with propriety include all shades of position as well here as in any other presentation; for a very small difference in the pelvic slopes will sometimes cause a vast modification of a vertex presentation. Any ordinary obstetrician can, after a few hours of impatient waiting, discover some kind of a misfit between the outline of the fœtal head and the beautifully described pelvic planes as set forth by Meigs and Bedford.

Obliquity of the uterus, according to most authors, is believed to be the cause of a very large majority of face presentations, from the fact that in nearly all cases, immediately after the diagnosis of face presentation is confirmed, obliquity of the womb is found to be present. Yet, frequently obliquity of the uterus is *well* marked, and the face does not present. We find recorded a number of face presentations observed during the post-mortem examinations, which we think prove very little, the diagnosis not having been made out previous to the death of the mother. If the mother died before the fœtus made its last effort to move, the absence of contractile power of the uterus would permit the child to assume almost any position, and remain there indefinitely. Again, the fœtus might gravitate, regardless of any and all surrounding circumstances, either with tendencies to bring about face presentation or any other head position. It seems reasonable to grant that in case of dead fœtus with head presenting the chin would drop away from the chest. We find all manner of presentations when the child is dead in utero, previous to setting in of labor pains. It is in such cases that a large quantity of liquor amnii is usually present, and the membranes usually protrude unobstructed, containing an excess of fluid. The membranes being ruptured, a large quantity of fluid suddenly escapes, thus accounting for a portion of the excess in number of face presentations, when the fœtus dies sometime before the beginning of labor. Another cause of face presentations: In dead children there is a lack of resistance on the part of the spinal column when the uterus is conducting the early stage of labor and the fundus pressure cannot be properly centred. In other words, the axis of the fœtus cannot be sustained; the mechanism of labor becomes unnatural, and the result doubtful. We might state that we believe any cause that may lead to the separation of the chin from the chest will lead to a face or brow presentation. This cause may exist in the fœtus itself or in the anatomical formation of the mother's pelvis. The bony or soft parts may be at fault. Ahlfeld ascribes one case to enlargement of the thyroid gland. Increased size of the chest; some unusual looping of the umbilical cord; either too small or too great a quantity of amniotic fluid may give rise to face presentation. The causes may be physiological, pathological, anatomical, and we might safely and truthfully state, traumatic or surgical, for not long since I heard a physician say that he believed he converted a crown to a face presentation. Hecker places great stress upon the shape of the child's head. Judging from the appearance of most of the babies that have faced me as an obstetrician, I would be perfectly willing to grant Hecker the full credit of his claim. Some of them were days in getting into human shape. The diagnosis may be very easy for some, but with others the task is a troublesome one. My first case was properly made out after some time. I have always congratulated myself on the case, believing that it was some unusual form of face presentation that never occurred before, and I am very certain have not made a similar diagnosis since.

By the touch only can the diagnosis be made, and then the membranes must not be thick or largely distended. You must see that everything connected with the case is favorable to an early diagnosis, for in the majority of cases the head remains high and the membranes low, and usually the frontal bone can most easily be touched, and it moves about with such a degree of facility that the obstetrician frequently finds it expedient to remain silent in the presence of the lady in labor and her near relatives. The outline of the face, according to most authorities, and all the obstetricians, is the only reliable sign. These, however, are easily determined as soon as the head descends low enough.

The mechanism of labor in face presentations is similar in nearly all respects to that of head in any other position. When the chin presents in either of the lateral positions, descent and rotation, though somewhat delayed, always take place without interference. It is when rotation of the chin does not take place that the skill of the obstetrician is taxed. That labors have terminated successfully without the rotation of the chin we cannot doubt, when such men as Partridge, Taylor, Tarriere and others report them. But in nearly all cases the results are disastrous.

I have succeeded in rotating the head three times, I think, in all; once in converting a face to a vertex. After repeated efforts the patient was directed to take the knee-chest position. I had not read of placing the patient in this position with a view of converting a face to a vertex, nor can I speak of it in the hands of others, but I accomplished the end without much difficulty by placing my left hand under the abdomen of my patient and introducing the right hand well into the vagina, and by upward pressure I was, with very gentle pressure against the head with index and middle fingers of right hand, enabled to force back the chin and face. I then placed the woman on her left side, and in a few minutes the vertex presented. I then ruptured the membranes. The case terminated successfully in a short time.

